# Early Identification of Low Bone Density Risk Using a Radiofrequency Echographic Multi Spectrometry-Based Prediction Model

**DOI:** 10.3390/life16071191

**Published:** 2026-07-18

**Authors:** Elena Bischoff, Stoyanka Vladeva, Nikola Kirilov, Fabian Bischoff

**Affiliations:** 1 Department of Health Care, Faculty of Medicine, Trakia University, 6000 Stara Zagora, Bulgaria; 2 Rheumazentrum Ruhrgebiet Herne, Ruhr-Universität Bochum, 44649 Herne, Germany; 3 Department of Orthopedics and Traumatology, University Hospital UMBAL Dr. Georgi Stranski, Medical University of Pleven, 5803 Pleven, Bulgaria; 4 Rheumatology Practice Stara Zagora, 6000 Stara Zagora, Bulgaria

**Keywords:** low bone mineral density, REMS, logistic regression, identification model, women

## Abstract

Osteoporosis is a major public health problem characterized by reduced bone mineral density (BMD) and increased fracture risk. Early identification of individuals with low bone density remains essential for prevention. This study aimed to evaluate a multivariable logistic regression-based model integrating clinical and Radiofrequency Echographic Multi Spectrometry (REMS)-derived parameters for the detection of low bone density in women. A total of 324 women undergoing REMS assessment of the lumbar spine and hip were included. Clinical variables (age, body mass index [BMI], menopausal status, and lifestyle factors) and REMS-derived measurements were analyzed. Binary logistic regression was used to identify independent factors associated with low BMD (T-score < −1 SD). Model performance was assessed using odds ratios (ORs), omnibus chi-square testing, pseudo-R^2^ statistics, and classification accuracy. The Youden index was applied to determine optimal cut-off values. Age, menopausal status, BMI, and basal metabolic rate (BMR) were identified as independent factors associated with low lumbar spine and femoral neck BMD. Increasing age and menopause were associated with higher odds of low bone density, whereas higher BMI and BMR were inversely associated with low bone density. In conclusion, a logistic regression model combining clinical and REMS-derived parameters demonstrated the ability to identify women with low bone density, supporting improved individualized risk stratification in clinical practice.

## 1. Introduction

Osteoporosis is a systemic skeletal disorder characterized by progressive loss of bone mass and deterioration of bone microarchitecture and quality, leading to increased skeletal fragility and a markedly elevated risk of low-energy fractures [1]. These fractures most commonly involve the vertebral bodies, proximal humerus, distal radius, and proximal femur and are associated with substantial morbidity, functional decline, loss of independence, and increased mortality [2].

From a public health perspective, osteoporosis represents a major and growing socioeconomic burden in aging populations. Annual costs related to osteoporotic fractures are substantial, reaching approximately 13 billion euros in Germany [3,4] and up to 19 billion US dollars in the United States [5]. Given the projected increase in life expectancy and aging-related fracture incidence, there is a critical need not only for early detection of low bone density but also for reliable approaches that allow individualized assessment of bone health status.

Fracture risk is inherently multifactorial and results from the interaction of non-modifiable and modifiable determinants. Established clinical prediction tools such as Fracture risk assessment tool (FRAX) [6] and the fracture risk calculator of the German Association of Orthopaedics and Trauma Surgery [7,8] integrate key variables including age, sex, body mass index, prior fragility fractures, smoking status, alcohol consumption, comorbidities, pharmacological exposures, and bone mineral density (BMD) [9,10]. Although these models are widely used, their predictive performance remains population-dependent and may not fully capture early metabolic or structural changes in bone tissue, particularly in individuals with borderline or subclinical disease.

Dual-energy X-ray absorptiometry (DXA) remains the reference standard for BMD assessment; however, its limitations include exposure to ionizing radiation, restricted accessibility in some clinical settings and limited sensitivity to early microarchitectural deterioration. These limitations highlight the need for complementary, non-invasive imaging modalities that can be integrated into clinical assessment frameworks for early identification of low bone density.

Radiofrequency Echographic Multi Spectrometry (REMS) is an emerging, radiation-free ultrasound-based technology for bone assessment [11]. Previous studies have demonstrated good agreement between REMS-derived measurements and DXA at clinically relevant skeletal sites, including the lumbar spine and proximal femur [12,13,14]. REMS is based on the analysis of backscattered radiofrequency ultrasound signals acquired from predefined regions of interest, which are processed using spectral analysis and compared with a validated reference database to estimate bone mineral density and derive a T-score [14,15].

Importantly, REMS provides not only quantitative bone density measurements but also additional parameters related to bone quality, including a fragility score. These complementary features make REMS particularly suitable for integration into multivariable assessment models, as they may capture aspects of bone strength not fully reflected by BMD alone [16].

However, despite its promising potential, REMS remains a relatively new technology and several aspects require further investigation. Current limitations include the need for additional validation in diverse populations, potential variability related to acquisition procedures and operator experience, and limited evidence regarding its incorporation into large-scale clinical assessment models. Further studies are needed to determine the generalizability and clinical utility of REMS-based approaches in identifying individuals with low bone density [17,18,19].

In 2026, REMS was included as a validated diagnostic modality in the recommendations of the Polish Society of Rheumatology, further supporting its clinical applicability and potential role in modern bone health assessment strategies [20].

Despite growing evidence supporting REMS, there is still limited knowledge regarding how REMS-derived parameters interact with classical clinical risk factors in multivariable models for identifying low bone density. In particular, few studies have applied multivariable logistic regression modeling to combine REMS-derived measurements with demographic and clinical variables for the purpose of identifying women with low bone density. This gap is clinically relevant, as current approaches for osteoporosis assessment still have limitations. Although DXA is considered the reference method for bone mineral density evaluation, its requirement for dedicated equipment and trained personnel may limit its accessibility for widespread screening. Furthermore, FRAX estimates the probability of osteoporotic fractures based on clinical risk factors but does not directly assess bone density and is validated primarily for individuals aged 40 years and older. Consequently, there remains a need for accessible, radiation-free approaches that can facilitate earlier detection of low bone density and improve individualized risk stratification. REMS represents a promising alternative technique for this purpose; however, prediction models integrating REMS-derived parameters with clinical factors remain insufficiently explored. We hypothesized that combining REMS-derived parameters with demographic and clinical risk factors would improve the identification of women with low bone density compared with the assessment of individual risk factors alone. Therefore, the aim of this study was to develop and evaluate a multivariable logistic regression-based model incorporating clinical, demographic and REMS-derived parameters for the identification of low bone density in women.

## 2. Materials and Methods

This was a prospective observational study including a total of 324 women who underwent REMS assessment. Participants were recruited from June 2023 to January 2026, signing an informed consent form prior to participation. Subjects were assessed at two centers—Trakia University and a rheumatology practice in Stara Zagora, Bulgaria. The study protocol was approved by the ethics committee of Trakia University, Stara Zagora, Bulgaria. Of the 324 women included in the study, 273 had complete data for all variables included in the logistic regression analysis and were therefore included in the final model. Participants with missing relevant variables were excluded from the regression analysis, and no imputation methods were applied.

Study population

Inclusion criteria comprised women referred for BMD assessment who underwent REMS evaluation of the lumbar spine and/or hip region and had complete clinical and densitometric datasets. Exclusion criteria included incomplete clinical or imaging data, missing risk factor information required for FRAX calculation, and technically inadequate scans.

Clinical assessment

A standardized clinical evaluation was performed prior to REMS scanning. Recorded variables included age, height, weight, BMI (automatically calculated as kg/m^2^), age at menopause, prior fracture history (low-trauma fractures, including morphometrically confirmed vertebral fractures), parental hip fracture, smoking status, and alcohol intake (≥3 units/day; 1 unit ≈ 8–10 g ethanol). Corticosteroid exposure was defined as current or prior oral glucocorticoid therapy for ≥3 months at a dose equivalent to ≥5 mg/day prednisone. Rheumatoid arthritis was included only if diagnosed according to established criteria, while osteoarthritis was not considered a risk factor. Secondary osteoporosis was defined as osteoporosis associated with type 1 diabetes mellitus, osteogenesis imperfecta in adults, untreated hyperthyroidism, hypogonadism or premature menopause (<45 years), chronic malnutrition/malabsorption, or chronic liver disease.

REMS measurements

REMS examinations were performed using the EchoStudio echographic device (Echolight S.p.a., Lecce, Italy), equipped with a convex transducer operating at a central frequency of 3.5 MHz. The operator performing the REMS examinations was blinded to the participants’ clinical characteristics and risk classification during image acquisition and analysis. Measurements were performed according to the manufacturer’s standardized acquisition protocol. All REMS examinations were performed by a single operator The same REMS device was used for all examinations performed at both study centers.

During examination, the transducer was positioned transabdominally for lumbar spine assessment and over the hip region for femoral measurements to visualize the predefined regions of interest (ROIs). After acquisition of an adequate B-mode ultrasound image, the REMS software (version 2.2.1) automatically identified the bone interfaces at the target skeletal sites. Ultrasound frame sequences were continuously recorded for approximately 80 s for the lumbar spine and 40 s for the proximal femur, allowing automated identification and analysis of the ROI.

Unlike conventional ultrasound imaging based only on B-mode images, REMS analyzes raw backscattered radiofrequency ultrasound signals. The software applies automated signal-processing algorithms to identify and exclude non-representative signals caused by artifacts. After artifact rejection, the remaining cortical and trabecular bone signals are processed to generate a patient-specific spectral profile. This spectral analysis is compared with a validated reference database to estimate BMD and derive T-scores and Z-scores. REMS-derived parameters included lumbar spine BMD (L1–L4), total lumbar spine BMD, T-score, and Z-score. Hip assessments included femoral neck, trochanteric and total hip BMD, T-scores, and Z-scores for the left and/or right sides when available.

Fracture risk assessment tool (FRAX) and body composition

In a subset of patients, 10-year fracture risk for major osteoporotic fracture (MOF) and hip fracture (HF) was calculated using the integrated FRAX tool version 1.4.4 (University of Sheffield algorithm). FRAX was calculated for women aged 40–90 years using femoral neck BMD (left hip) and clinical risk factors. FRAX was calculated in a subset of participants to provide additional information on estimated 10-year fracture probability and to characterize the clinical profile of the study population. FRAX-derived variables were not included in the logistic regression models because the primary outcome was the identification of low bone density based on a T-score < −1 SD rather than fracture risk estimation.

Body composition was assessed using the optional body composition software module integrated into the EchoStudio REMS system during lumbar spine acquisition. The software automatically estimates body fat percentage (BF), basal metabolic rate (BMR), and body mass index (BMI). BF is calculated using a proprietary multiple regression algorithm that combines ultrasound-derived measurements of abdominal soft tissues, subcutaneous fat, and muscle thickness with patient characteristics (age, sex, height, and weight). The algorithm has been calibrated against bioelectrical impedance measurements. BMI was calculated as weight (kg) divided by height squared (m^2^), and BMR was estimated automatically by the EchoStudio software.

Statistical Analysis

Statistical analyses were performed using SPSS version 19.0 (IBM Corp., Armonk, NY, USA). Data were summarized using descriptive statistics (mean, standard deviation, standard error, minimum, and maximum).

Associations between categorical variables were evaluated using the chi-square test. Group comparisons were performed using Student’s *t*-test after assessment of normality and homogeneity of variance (Levene’s test); otherwise, the Mann–Whitney U test was applied. One-way ANOVA was used for comparisons among more than two groups.

Binary logistic regression analysis was performed to identify independent factors associated with low bone density, defined as a T-score < −1 SD. The primary outcome was defined as low bone density, corresponding to a T-score < −1 SD. According to established diagnostic criteria, a T-score between −1.0 and −2.5 SD indicates low bone density (osteopenia), whereas osteoporosis is defined as a T-score ≤ −2.5 SD. Therefore, the term “low bone density” is used throughout the manuscript to describe the study outcome. The dependent variable was dichotomized as normal bone density (T-score ≥ −1 SD) versus low bone density (T-score < −1 SD). Participants with a T-score exactly equal to −1 SD were classified as having normal bone density. Candidate predictor variables included age, weight, height, BMI, menopausal status, body fat percentage, and basal metabolic rate. Variables significantly associated with the outcome in univariate analyses were entered into a multivariable binary logistic regression model. A forward conditional stepwise selection procedure was applied. In this approach, predictor variables were entered into the model sequentially based on their statistical contribution, with variables meeting the entry criterion added to the model and variables failing to meet the retention criterion removed during subsequent steps. The final model included only variables that remained independently associated with low bone density. Odds ratios (ORs) with 95% confidence intervals (CIs) were calculated for each predictor. Model performance was assessed using the omnibus chi-square test and pseudo-R^2^ statistics (Cox and Snell, and Nagelkerke). Complete-case analysis was performed for the regression models. Participants with missing values for variables required for the analysis were excluded from the regression analyses, and no imputation methods were applied. Of the 324 women included in the study, 273 had complete data for all variables included in the final regression model and were therefore included in the analysis.

The Youden index (sensitivity + specificity − 1) was used to determine optimal cut-off values, with higher values indicating better discriminative performance for identifying low bone density.

The reporting of this multivariable logistic regression analysis was guided by the principles outlined in the Transparent Reporting of a multivariable prediction model for Individual Prognosis Or Diagnosis (TRIPOD) statement. Potential methodological limitations related to model applicability and risk of bias were considered in accordance with the domains described in the Prediction model Risk Of Bias ASsessment Tool (PROBAST) [21,22].

## 3. Results

The mean age of the study population was 62 ± 12 years (range 25–88 years). BMI differed across diagnostic groups: 32.4 ± 6.4 kg/m^2^ in women with normal lumbar spine BMD (20.4–47.5 kg/m^2^), 28.3 ± 5.6 kg/m^2^ in osteopenia (16.9–46.4 kg/m^2^), and 25.9 ± 4.9 kg/m^2^ in osteoporosis (14.9–37.7 kg/m^2^). A total of 260 women (80%) were postmenopausal, with a mean age at menopause of 47 ± 5 years (33–56 years). Premature menopause (<45 years) was present in 70 women (21.6%). Among postmenopausal women, osteopenia was most frequent (51.9%), followed by osteoporosis (35.4%) and normal BMD (12.7%). In premenopausal women, normal BMD predominated (62.5%), followed by osteopenia (34.4%) and osteoporosis (3.1%). Among the 304 women with complete FRAX-related data, 107 (35.2%) had a history of prior fracture, 10 (3.3%) reported parental hip fracture, 69 (22.7%) were current smokers and 20 (6.6%) reported alcohol intake >3 units/day. Rheumatoid arthritis was present in 68 (22.4%), and 39 (12.8%) were receiving corticosteroids. Secondary osteoporosis due was present in 70 women (23%).

Lumbar spine BMD values were L1 (0.765 ± 0.152 g/cm^2^), L2 (0.837 ± 0.145 g/cm^2^), L3 (0.892 ± 0.137 g/cm^2^), and L4 (0.918 ± 0.137 g/cm^2^). Total lumbar spine BMD was 0.860 ± 0.140 g/cm^2^. Mean T-score was −1.7 ± 1.2 SD and Z-score −0.2 ± 0.9 SD. Mean body fat percentage was 37.8 ± 8.8%, and mean BMR was 1274.0 ± 163.2 kcal/day (Table 1).

The mean BMD of the left femoral neck was 0.671 ± 0.133 g/cm^2^ (range 0.347–1.093 g/cm^2^). The corresponding T-score was −1.7 ± 1.1 SD (range −4.5 to 2.2 SD), and the Z-score was −0.2 ± 1.0 SD (range −2.4 to 3.3 SD). For the left trochanter, mean BMD was 0.833 ± 0.142 g/cm^2^ (range 0.447–1.316 g/cm^2^), with a T-score of −0.8 ± 1.0 SD (range −3.6 to 2.9 SD) and a Z-score of −0.1 ± 0.9 SD (range −2.1 to 3.5 SD). Total left hip BMD was 0.818 ± 0.149 g/cm^2^ (range 0.433–1.275 g/cm^2^), with a total T-score of −1.1 ± 1.1 SD (range −4.2 to 2.7 SD) and a Z-score of 0 ± 1.0 SD (range −2.2 to 3.5 SD). The mean 10-year FRAX risk for major osteoporotic fracture (MOF), calculated using the left femoral neck T-score, was 15.5% ± 11.6% (range 1.9–66.9%), while the corresponding hip fracture risk was 4.7% ± 7.3% (range 0.0–56.2% (Table 2).

A stepwise binary logistic regression analysis was performed to determine which variables represent independent risk factors for lumbar spine (LS) BMD corresponding to a T-score < −1 SD. Two groups were defined: one including women with an LS T-score > −1 SD and the other including women with an LS T-score < −1 SD. The regression model included all significant risk factors in the univariate analysis: age, weight, height, BMI, menopause status, body fat composition, and basal metabolic rate (BMR).

The binary logistic regression was conducted in four steps, with the final step used to identify independent significant predictors of LS T-score < −1 SD. According to the classification table, the model was based on 273 of 324 women (84.3%). The overall predictive accuracy was 94.9%, correctly classifying 82.7% of women with T-score > −1 SD and 97.7% of women with T-score < −1 SD. The model was statistically significant at Step 4 (*p* = 0.014). Table 3 presents the results of the final step of the binary logistic regression analysis.

From stepwise binary logistic regression analysis, we found that the independent risk factors significantly associated with low lumbar spine BMD corresponding to a T-score < −1 SD were age, BMI, menopause status, and basal metabolic rate (BMR). Weight and height were excluded in the initial steps of the regression model, most likely because they are not independent of BMI as a risk factor for T-score < −1 SD. Similarly, body fat percentage was excluded in the early steps, as it is not independent of BMR as a risk factor for T-score < −1 SD.

After calculating the odds ratios for the independent factors, we found that each additional year of age was associated with a 15.5% increase in the odds of having a lumbar spine T-score < −1 SD (OR = 1.155). More precisely, after assessing model sensitivity, specificity, and the corresponding Youden indices, we identified 65 years as the optimal cut-off value for low lumbar spine BMD corresponding to a T-score < −1 SD. This indicates that women older than 65 years have a higher probability of presenting with a lumbar spine T-score below −1 SD.

The odds ratio for menopause as a factor associated with a T-score < −1 SD was 9.54, indicating that postmenopausal women had approximately 9.5-fold higher odds of low lumbar spine BMD compared with premenopausal women.

The odds ratio for BMI was 0.85, indicating an inverse association. Specifically, each 1 kg/m^2^ increase in BMI was associated with an approximately 15.4% decrease in the odds of having a T-score < −1 SD (OR = 0.846). Based on model sensitivity, specificity, and Youden index analysis, women with a BMI > 28.63 kg/m^2^ are less likely to have a lumbar spine T-score < −1 SD.

**Figure 1:**
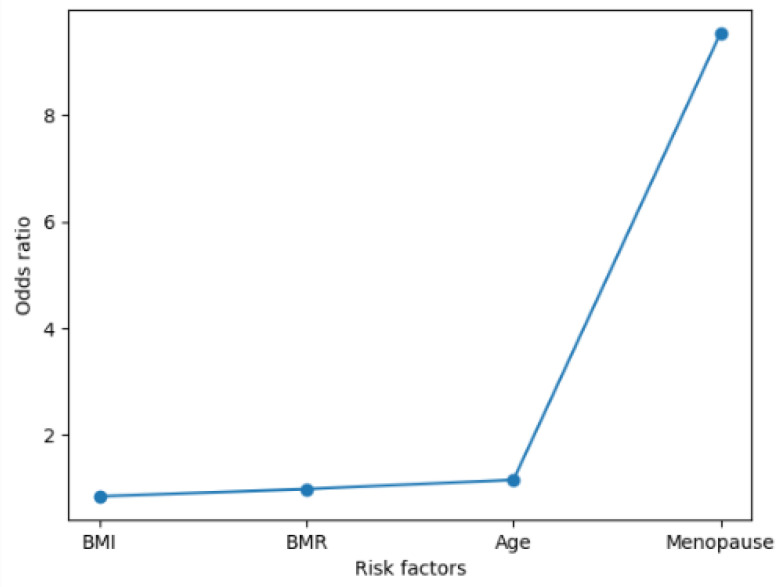
Graphical representation of odds ratios of the independent risk factors for a T-score < −1 SD of the lumbar spine.

The odds ratio for BMR was 0.982, also indicating an inverse association. Each increase of 1 kcal/day in BMR was associated with a 1.8% decrease in the odds of having a T-score < −1 SD (OR = 0.982). Accordingly, women with a BMR > 1331.75 kcal/day are less likely to present with low lumbar spine BMD corresponding to a T-score < −1 SD. All odds ratios are graphically presented in Figure 1.

The model demonstrated good discrimination, with an area under the curve (AUC) of 0.82 (95% CI: 0.76–0.88). The optimal cut-off value was identified using the maximum Youden index, achieving a sensitivity of 94.9% and specificity of 82.7%. ROC curve analysis identified optimal cut-off values of 65 years for age, 28.63 kg/m^2^ for BMI, and 1331.75 kcal/day for BMR.

After determining, using the univariate ANOVA test, which risk factors differ significantly between the groups with normal BMD of the left hip, osteopenia and osteoporosis of the left femoral neck, we performed a stepwise binary regression analysis to identify which of them are independent risk factors for left femoral neck BMD corresponding to a T-score < −1 SD. We created two groups: one consisting of women with a left hip T-score > −1 SD, and the other consisting of women with a left hip T-score < −1 SD. The regression analysis included all significant risk factors—age, weight, height, BMI, menopause status, body fat composition, and basal metabolic rate.

The stepwise binary regression analysis was conducted in four steps, with the fourth step used to assess which risk factors remained independent significant predictors of a left hip T-score < −1 SD. According to the resulting classification table, the binary regression analysis was performed on 273 of 324 women (84.3%). The model correctly classified 94.9% of cases, with 82.7% correctly classified in women with a T-score above −1 SD and 97.7% correctly classified in women with a T-score below −1 SD. Overall, the model was statistically significant (*p* = 0.014) at the fourth step (Table 4).

From the stepwise binary logistic regression analysis, we identified age, BMI, menopause status and basal metabolic rate (BMR) as independent risk factors significantly associated with low bone mineral density of the left femoral neck corresponding to a T-score < −1 SD. Weight and height were excluded in the early steps of the model, most likely due to their lack of independence from BMI as a risk factor for a T-score < −1 SD of the left femoral neck, while body fat composition was excluded in the initial steps, most likely due to its dependency on BMR. After calculation of odds ratios (ORs) for the independent predictors, each additional year of age was associated with an approximately 18% increase in the odds of having a T-score < −1 SD at the left femoral neck (OR = 1.180). Based on sensitivity and specificity analyses and corresponding Youden indices, 63 years was identified as a threshold value for low bone mineral density at the left femoral neck. This indicates that increasing age was associated with a higher likelihood of presenting with a T-score < −1 SD.

Menopause was associated with a 16.6-fold increase in the odds of having a T-score < −1 SD at the left femoral neck compared with premenopausal women (OR = 16.6). The OR for BMI was 0.65, indicating an inverse association; each 1 kg/m^2^ increase in BMI was associated with an approximately 35% decrease in the odds of having a T-score < −1 SD at the left femoral neck (OR = 0.65). More precisely, threshold analysis showed that women with a BMI > 26.5 kg/m^2^ were less likely to have a T-score < −1 SD at the left femoral neck.

**Figure 2:**
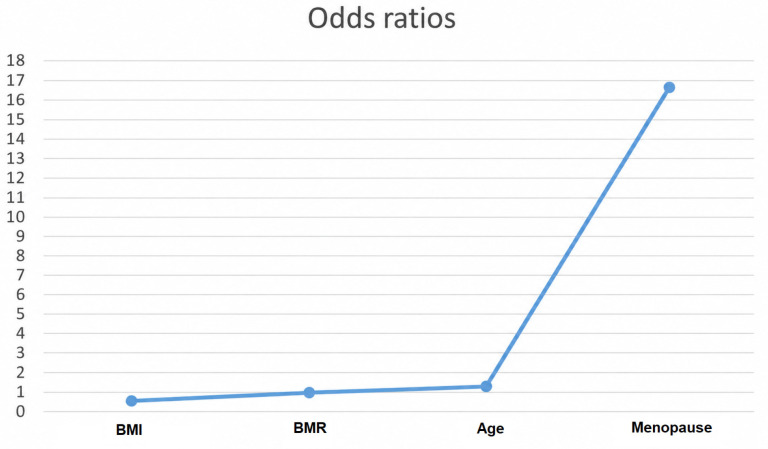
Graphical representation of the odds ratios of the independent risk factors for a T-score of the left femoral neck < −1 SD.

The OR for BMR was 0.98, indicating an inverse association. Each additional 1 kcal/day increase in BMR was associated with an approximately 2% decrease in the odds of having a T-score < −1 SD (OR = 0.98). Given the small unit of measurement, this association should be interpreted in relation to overall differences in BMR rather than single kcal/day increments. A threshold value of >1364.4 kcal/day was associated with lower odds of low bone mineral density at the left femoral neck (Figure 2).

ROC curve analysis demonstrated good discriminative performance of the model for identifying low bone mineral density at the left femoral neck, with an AUC of 0.85 (95% CI: 0.79–0.90), indicating a high ability to distinguish between women with and without low bone mineral density. The optimal cut-off value was determined using the maximum Youden index, achieving a sensitivity of 94.9% of cases and specificity of 82.7%. ROC analysis identified optimal cut-off values of 63 years for age, 26.5 kg/m^2^ for BMI, and 1364.4 kcal/day for BMR.

**Figure 3:**
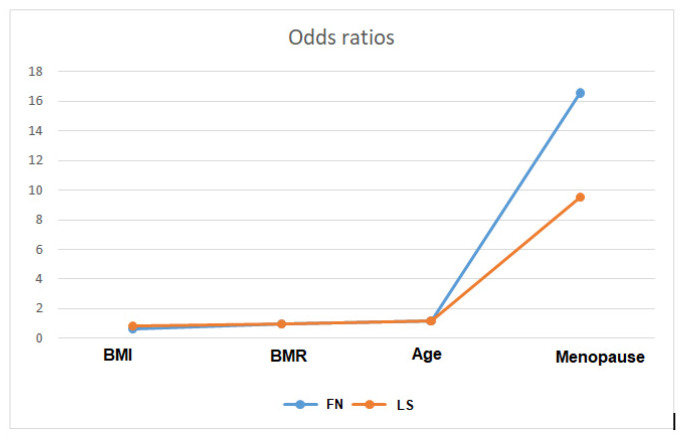
Graphical representation of odds ratios of the independent risk factors for a T-score < −1 SD of the hip and lumbar spine.

When comparing the two models, we find that the independent risk factors BMI, BMR, and age have a relatively similar predictive ability for a T-score < −1 SD, both for the left hip and the lumbar spine. Only the independent risk factor menopause shows approximately a twofold higher risk of a T-score < −1 SD in the left hip compared to a T-score < −1 SD in the lumbar spine (Figure 3).

## 4. Discussion

To our knowledge, few studies have investigated factors associated with low bone density using REMS-derived parameters. The present study contributes additional evidence by evaluating the association between clinical characteristics and REMS-based measurements in women with different bone density profiles. REMS is an innovative, non-ionizing technology that enables assessment of bone quantity through BMD, T-score, and Z-score measurements, while also providing additional information related to bone quality through parameters such as the fragility score. By analyzing raw radiofrequency ultrasound signals, REMS provides accurate and reproducible measurements at clinically relevant skeletal sites, while avoiding some limitations of conventional densitometry, including ionizing radiation exposure, artifacts, and limited portability. These characteristics support the potential role of REMS as a complementary tool for bone assessment, monitoring, and individualized management strategies in different patient populations [23]. Of the 324 women included, 260 were postmenopausal and 64 premenopausal. Among the postmenopausal women, 51.9% had low bone density (osteopenia range), 35.4% had osteoporosis-range T-scores, and 12.7% had normal bone mineral density. In the premenopausal group, 34.4% had low bone density, 3.1% had osteoporosis-range T-scores, and 62.5% had normal bone mineral density. Similar findings were reported by John Cecily et al., who examined 200 women and found osteopenia in 63% of postmenopausal women and 36% of premenopausal women [24].

Our results from binary logistic regression identified independent factors associated with low bone density in both the lumbar spine and proximal femur. The independent factors associated with low bone density at the lumbar spine were age (*p* < 0.001) with an odds ratio of 1.155, BMI (*p* = 0.015) with an odds ratio of 0.846, and menopause (*p* = 0.006) with an odds ratio of 9.538. Basal metabolic rate also showed a significant association with low bone density at the lumbar spine, with an odds ratio of 0.982 (*p* < 0.001).

The independent factors associated with low bone density at the proximal femur were age (*p* = 0.000) with an odds ratio of 1.179, BMI (*p* = 0.000) with an odds ratio of 0.646, menopause (*p* = 0.003) with an odds ratio of 16.569, and BMR (*p* = 0.000) with an odds ratio of 0.983.

To date, no other study has investigated factors associated with low bone density using the REMS method. Previous publications have focused mainly on fracture risk assessment using REMS [16,17,25].

Most previous studies have focused on risk factors associated with osteoporotic fractures [26,27]. Kanis and McCloskey described risk factors associated with osteoporosis in 1998, identifying a wide range of factors including genetic predisposition, ethnicity, family history, early menopause, late menarche, lifestyle factors, smoking and alcohol consumption, physical inactivity, immobilization, low BMI, various diseases, and medications [28]. However, they did not provide a detailed weighting of these factors. These factors are consistent with those identified in our study using the REMS method.

Aloia et al. investigated factors associated with osteoporosis in 1985, focusing on laboratory parameters and demonstrating the relevance of estrogen, testosterone, alkaline phosphatase, and vitamin D. In addition, smoking, body weight, and height were identified as further factors associated with bone status [29].

Several authors have already addressed the question of osteoporosis assessment and classification. The majority of these studies used artificial intelligence (AI), deep learning (DL), or machine learning (ML). Wu and Park developed a machine learning model for identifying osteoporosis, analyzing genetic factors, number of children and breastfed children, age, residential area, education, season of measurement, height, body weight, smoking, hormone replacement therapy, serum albumin, hip circumference, and vitamin B6 intake. They also found an association of age and low energy levels with osteoporosis status [30].

The meta-analysis by Amani et al. evaluated the diagnostic accuracy of deep learning for osteoporosis detection. They demonstrated that deep learning represents a promising approach; however, most of the included studies were based on the analysis of radiographic images [31].

Strengths and limitations

This study has several important strengths. First, it addresses a clinically relevant topic by evaluating factors associated with low bone density using REMS, a radiation-free and non-invasive technology with potential advantages for accessible bone health assessment. Second, this study integrates REMS-derived parameters with clinical characteristics, allowing evaluation of the combined contribution of imaging-based and patient-related factors in identifying women with low bone density. Third, this study includes a relatively large cohort of 324 women assessed using a standardized REMS acquisition protocol, with examinations performed using the same device and by the same experienced operator, which reduces variability related to technical procedures. Fourth, the statistical approach included multivariable logistic regression analysis, evaluation of odds ratios with confidence intervals, and assessment of model discrimination using ROC analysis and Youden index-based cut-off determination.

Despite the promising findings, several limitations should be acknowledged. First, this study included both premenopausal and postmenopausal women in a single analytical cohort. Since T-scores are not routinely recommended for premenopausal women, according to ISCD guidelines, their inclusion may have introduced heterogeneity and limited applicability to younger women. Future studies should validate the model separately in premenopausal and postmenopausal populations using appropriate densitometric criteria.

Second, participants were recruited from a specialized setting and were referred for bone density evaluation rather than selected from a general population-based sample. This may have resulted in a higher proportion of women with factors associated with low bone density, including rheumatoid arthritis, potentially introducing selection bias and affecting estimates of model performance. Therefore, validation in independent population-based cohorts is required to confirm external validity. Third, DXA measurements were not available, preventing direct comparison of the REMS-based model with the current reference standard for BMD assessment. Although REMS measurements were performed according to a standardized protocol, variations related to operator performance and technical factors cannot be completely excluded. Furthermore, the use of a stepwise regression approach may increase the risk of model overfitting, and some potential confounding variables may not have been fully adjusted for. Finally, internal and external validation of the identification model were not performed. Future studies including appropriate validation methods and independent cohorts are necessary to confirm the reproducibility, generalizability, and clinical applicability of the findings.

## 5. Conclusions

Previous studies on osteoporosis assessment and fracture risk estimation have mainly relied on laboratory diagnostics and DXA-based measurements. New approaches using computer-assisted analyses primarily focus on image-based assessment. Our study identifies independent factors associated with low bone density determined by the REMS method, with three of four factors being obtainable entirely without laboratory or imaging-based diagnostics. When combined with REMS, these factors provide additional information alongside REMS-derived T-scores and basal metabolic rate, supporting improved identification of women with low bone density within the studied cohort.

However, as this cross-sectional model was developed using a single cohort without external validation or direct comparison with established methods, further studies in independent populations are required to confirm its reproducibility, generalizability, and potential clinical applicability.

## Figures and Tables

**Table 1:** Values obtained from REMS measurements of the lumbar spine.

REMS Measurements of LS	Mean Value	Min Value	Max Value	SD	SE
BMD L1	0.765	0.427	1.235	0.152	0.008
BMD L2	0.837	0.567	1.310	0.145	0.008
BMD L3	0.892	0.578	1.303	0.137	0.008
BMD L4	0.918	0.632	1.341	0.137	0.008
Total BMD LS	0.860	0.529	1.291	0.140	0.008
Total T-score LS	−1.7	−4.7	3.0	1.2	0.1
Total Z-score LS	−0.2	−2.0	3.1	0.9	0.0
Fat (%)	37.8	9.0	52.0	8.8	0.5
BMR (kcal/day)	1274.0	929.7	1908.4	163.2	9.8

**Table 2:** Values obtained from REMS measurements of the left hip.

REMS Measurements of the Left Hip	Mean Value	Min Value	Max Value	SD	SE
BMD femoral neck (FN)	0.671	0.347	1.093	0.133	0.008
T-score FN	−1.7	−4.5	2.2	1.1	0.1
Z-score FN	−0.2	−2.4	3.3	1.0	0.1
BMD trochanter	0.833	0.447	1.316	0.142	0.008
T-score trochanter	−0.8	−3.6	2.9	1.0	0.1
Z-score trochanter	0.1	−2.1	3.5	0.9	0.1
Total hip BMD	0.818	0.433	1.275	0.149	0.009
Total hip T-score	−1.1	−4.2	2.7	1.1	0.1
Total hip Z-score	0.0	−2.2	3.5	1.0	0.1
FRAX MOF	15.5	1.9	66.9	11.6	0.7
FRAX HF	4.7	0.0	56.2	7.3	0.4

**Table 3:** Parameters from the fourth step of the binary logistic regression analysis for prediction of LS T-score < −1 SD.

Independent Risk Factors	B (Regression Coefficient)	SE	Wald	df	*p*-Value	Odds Ratio	95% Confidence Interval for OR
Age	0.144	0.039	13.462	1	<0.001	1.155	1.070–1.247
BMI	−0.167	0.069	5.976	1	0.015	0.846	0.739–0.969
Menopause	2.255	0.819	7.586	1	0.006	9.538	1.915–47.470
Basal metabolic rate (BMR)	−0.019	0.004	26.904	1	<0.001	0.982	0.973–0.989
Constant	21.291	4.124	26.659	1	<0.001	1.764 × 10^9^	

**Table 4:** Results from the binary regression analysis for the prediction of left femoral neck T-score < −1 SD.

Independent Risk Factors	B	SE	Wald	df	*p*-Value	OR	95% Confidence Interval for OR
Step 4							
Age	0.165	0.043	14.884	1	0.000	1.179	1.084–1.282
BMI	−0.437	0.098	20.010	1	0.000	0.646	0.533–0.782
Menopause	2.808	0.952	8.702	1	0.003	16.569	2.551–107.557
BMR	−0.017	0.004	21.090	1	0.000	0.983	0.976–0.991
Constant	24.643	4.531	29.581	1	0.000	5.038 × 10^10^	

## Data Availability

The authors confirm that the data supporting the findings of this study are not publicly available due to privacy and ethical restrictions.

## References

[1] Kanis J.A. (2002). Diagnosis of osteoporosis and assessment of fracture risk. Lancet.

[2] Johnell O., Kanis J. (2005). Epidemiology of osteoporotic fractures. Osteoporos. Int..

[3] Kanis J.A., Johnell O., Oden A., Sernbo I., Redlund-Johnell I., Dawson A., De Laet C., Jonsson B. (2000). Long-Term Risk of Osteoporotic Fracture in Malmö. Osteoporos. Int..

[4] https://www.osteoporose-deutschland.de/osteoporose/daten-fakten/.

[5] Burge R., Dawson-Hughes B., Solomon D.H., Wong J.B., King A., Tosteson A. (2007). Incidence and economic burden of osteoporosis-related fractures in the United States, 2005–2025. J. Bone Miner. Res..

[6] Kanis J.A., Johansson H., Harvey N.C., McCloskey E.V. (2018). A brief history of FRAX. Arch. Osteoporos..

[7] Drey M., Otto S., Thomasius F., Schmidmaier R. (2024). Update der S3-Leitlinie Diagnostik, Prophylaxe und Therapie der Osteoporose. Die Orthop..

[8] Thomasius F., Kurth A., Baum E., Drey M., Maus U., Schmidmaier R. (2025). The Diagnosis and Treatment of Osteoporosis. Dtsch. Ärzteblatt Int..

[9] https://www.fraxplus.org/de/calculation-tool.

[10] https://osteoporose.bvou.net/.

[11] Fuggle N.R., Reginster J.Y., Al-Daghri N., Bruyere O., Burlet N., Campusano C., Brandi M.L. (2024). Radiofrequency echographic multi spectrometry (REMS) in the diagnosis and management of osteoporosis: State of the art. Aging Clin. Exp. Res..

[12] Liu R.Y., Liu E. (2026). Correlation between radiofrequency echographic multi-spectrometry (REMS) and dual-energy X-ray absorptiometry (DXA): A systematic review and meta-analysis. Bone.

[13] Caffarelli C., Pitinca M.D.T., Nuti R., Gonnelli S. (2019). Radiofrequency echographic multispectrometry (REMS): A new approach for osteoporosis diagnosis in adolescents. Bone Abstr..

[14] Di Paola M., Gatti D., Viapiana O., Cianferotti L., Cavalli L., Caffarelli C., Rossini M. (2019). Radiofrequency echo-graphic multispectrometry compared with dual X-ray absorptiometry for osteoporosis diagnosis on lumbar spine and femoral neck. Osteoporos. Int..

[15] Conversano F., Franchini R., Greco A., Soloperto G., Chiriacò F., Casciaro E., Casciaro S. (2015). A novel ultrasound methodology for estimating spine mineral density. Ultrasound Med. Biol..

[16] Pisani P., Conversano F., Muratore M., Adami G., Brandi M.L., Caffarelli C., Casciaro S. (2023). Fragility Score: A REMS-based indicator for the prediction of incident fragility fractures at 5 years. Aging Clin. Exp. Res..

[17] Icătoiu E., Vlădulescu-Trandafir A.I., Groșeanu L.M., Berghea F., Cobilinschi C.O., Potcovaru C.G., Bălănescu A.R., Bojincă V.C. (2025). Radiofrequency Echographic Multi Spectrometry-A Novel Tool in the Diagnosis of Osteoporosis and Prediction of Fragility Fractures: A Systematic Review. Diagnostics.

[18] Cortet B., Dennison E., Diez-Pérez A., Locquet M., Muratore M., Nogues X., Crespo D.O., Quarta E., Brandi M.L. (2021). Radiofrequency Echographic Multi Spectrometry (REMS) for the diagnosis of osteoporosis in a European multicenter clinical context. Bone.

[19] Diez-Perez A., Brandi M.L., Al-Daghri N., Branco J.C., Bruyère O., Cavalli L., Cooper C., Cortet B., Dawson-Hughes B., Dimai H.P. (2019). Radiofrequency echographic multi-spectrometry for the in-vivo assessment of bone strength: State of the art—Outcomes of an expert consensus meeting organized by the European Society for Clinical and Economic Aspects of Osteoporosis, Osteoarthritis and Musculoskeletal Diseases (ESCEO). Aging Clin. Exp. Res..

[20] Leszczyński P., Iwaszkiewicz C., Żuber Z.M., Kwiatkowska B., Batko B.P., Pawlak-Buś K.K. (2026). Diagnosis and treatment of osteoporosis: 2026 recommendations of the Polish Society for Rheumatology. Reumatologia.

[21] Collins G.S., Reitsma J.B., Altman D.G., Moons K.G. (2015). Transparent reporting of a multivariable prediction model for individual prognosis or diagnosis (TRIPOD): The TRIPOD statement. BMJ.

[22] Wolff R.F., Moons K.G.M., Riley R.D., Whiting P.F., Westwood M., Collins G.S., Reitsma J.B., Kleijnen J., Mallett S., PROBAST Group (2019). PROBAST: A Tool to Assess the Risk of Bias and Applicability of Prediction Model Studies. Ann. Intern Med..

[23] Ruggiero M., Iannotti F., Cava G., Floccari F., Chiappetta A., Bonifacio M., Iorio R., Conforti A. (2025). Radiofrequency echographic multi spectrometry technology: A new frontier to bone density assessment. Minerva Orthop..

[24] Cecily H.S.J. (2020). Early detection and prevention of osteoporosis among pre-and postmenopausal women in Saudi Arabia. Clin. Nurs. Res..

[25] Adami G., Arioli G., Bianchi G., Brandi M.L., Caffarelli C., Cianferotti L., Quarta L. (2020). Radiofrequency echo-graphic multi spectrometry for the prediction of incident fragility fractures: A 5-year follow-up study. Bone.

[26] De Laet C.E.D.H., Kanis J.A., Odén A., Johanson H., Johnell O., Delmas P., Tenenhouse A. (2005). Body mass index as a predictor of fracture risk: A meta-analysis. Osteoporos. Int..

[27] Johansson H., Kanis J.A., Odén A., McCloskey E., Chapurlat R.D., Christiansen C., Zillikens M.C. (2014). A meta-analysis of the association of fracture risk and body mass index in women. J. Bone Miner. Res..

[28] Kanis J.A., McCloskey E.V. (1998). Risk factors in osteoporosis. Maturitas.

[29] Aloia J.F., Cohn S.H., Vaswani A., Yeh J.K., Yuen K., Ellis K. (1985). Risk factors for postmenopausal osteoporosis. Am. J. Med..

[30] Wu X., Park S. (2023). A prediction model for osteoporosis risk using a machine-learning approach and its validation in a large cohort. J. Korean Med. Sci..

[31] Amani F., Amanzadeh M., Hamedan M., Amani P. (2024). Diagnostic accuracy of deep learning in prediction of osteo-porosis: A systematic review and meta-analysis. BMC Musculoskelet. Disord..

